# Preterm birth and its associated factors among reproductive aged women in sub-Saharan Africa: evidence from the recent demographic and health surveys of sub-Sharan African countries

**DOI:** 10.1186/s12884-021-04233-2

**Published:** 2021-11-15

**Authors:** Tesfa Sewunet Alamneh, Achamyeleh Birhanu Teshale, Misganaw Gebrie Worku, Zemenu Tadesse Tessema, Yigizie Yeshaw, Getayeneh Antehunegn Tesema, Alemneh Mekuriaw Liyew, Adugnaw Zeleke Alem

**Affiliations:** 1 Department of Epidemiology and Biostatistics, Institute of Public Health, College of Medicine and Health Sciences, University of Gondar, Gondar, Ethiopia; 2 Department of Human Anatomy, University of Gondar, College of Medicine and Health Science, School of Medicine, Gondar, Ethiopia; 3 Department of Physiology, School of Medicine, College of Medicine and Health Sciences, University of Gondar, Gondar, Ethiopia

**Keywords:** Preterm birth, Premature birth, Reproductive age women, Sub-Saharan Africa, Demographic and health survey, Multilevel analysis

## Abstract

**Background:**

Globally, preterm birth is the leading cause of neonatal and under-five children mortality. Sub-Saharan African (SSA) accounts for the majority of preterm birth and death following its complications. Despite this, there is limited evidence about the pooled prevalence and associated factors of preterm birth at SSA level using nation-wide representative large dataset. Therefore, this study aimed to determine the pooled prevalence and associated factors of preterm birth among reproductive aged women.

**Methods:**

The recent Demographic and Health Surveys (DHSs) data of 36 SSA countries were used. We included a total weighted sample of 172,774 reproductive-aged women who were giving birth within five years preceding the most recent survey of SSA countries were included in the analysis. We used a multilevel logistic regression model to identify the associated factors of preterm birth in SSA. We considered a statistical significance at a *p*-value less than 0.05.

**Results:**

In this study, 5.33% (95% CI: 5.23, 5.44%) of respondents in SSA had delivered preterm baby. Being form eastern Africa, southern Africa, rural area, being educated, substance use, having multiple pregnancy, currently working history, having history of terminated pregnancy, and previous cesarean section delivery, primi-parity, and short birth interval were associated with higher odds of preterm birth among reproductive aged women. However, having better wealth index, being married, wanted pregnancy, and having four or more antenatal care visit were associated with lower odds for a preterm birth among reproductive aged women.

**Conclusion:**

The prevalence of preterm birth among reproductive-aged women remains a major public health problem in SSA. Preterm birth was affected by various socio-economic and obstetrical factors. Therefore, it is better to consider the high-risk groups during intervention to prevent the short-term and long-term consequences of preterm birth.

**Supplementary Information:**

The online version contains supplementary material available at 10.1186/s12884-021-04233-2.

## Background

Preterm birth refers to the birth of a baby between 20 and 37 weeks of pregnancy [1]. It is the leading cause of death in children before the age of five years globally and accounts for one-third of the neonatal death [2]. Preterm birth is associated with serious short-term and long-term complications [3]. These include: respiratory distress syndrome [4, 5], bronchopulmonary dysplasia [4, 6], necrotizing enter-colitis [7, 8], neuro-developmental impairment (including intra-ventricular hemorrhage, and cerebral palsy) [3, 9, 10] chronic lung disease [11], hypertension [12, 13] and glucose intolerance [14].

From the 130 million babies born each year in the globe, approximately 15 million born too soon annually [15]. Over 60% preterm births and 80% of the world’s 1.1 million deaths due to preterm birth complications occur in South Asia and sub-Saharan Africa [16].

The finding of previous studies in the globe revealed that several factors are associated with preterm birth. These include: maternal age [17], wealth index [18], maternal education [19], residence [20], marital status [21], physical activity [22], mode of delivery [23], history of miscarriage or stillbirth [24], inter-pregnancy interval [25, 26], Ante Natal Care (ANC) [27, 28], substance use (cigarette and tobacco use) [29, 30], and multiple pregnancy [31, 32].

Despite preterm is associated with both short- and long-term consequences and majorly affects SSA which covers a significant portion of preterm birth global burden, to the best of our knowledge, the pooled prevalence and associated factors of preterm birth were not estimated at regional level that is Sub-Saharan Africa. The previous studies conducted on preterm birth in SSA countries were based on specific countries and were using small samples that are from facilities.

Therefore, this study aimed to investigate the pooled prevalence and associated factors of preterm birth among reproductive aged women who had given birth within five years preceding the DHS surveys in SSA countries. Conducting this will help to make decision on neonatal and child health based on best available scientific evidences. Moreover, the result of this study could support policy makers, clinicians, and programmers to design intervention on preventing preterm birth and its complications in the region.

## Methods

### Data source and sampling procedure

We used the most recent Demographic and Health Survey (DHS) datasets of 36 SSA countries conducted from 2006 to 2018 to determine the pooled prevalence and associated factors of preterm birth among reproductive aged women in SSA.

The DHS surveys are routinely collected every five-year across low-and middle-income countries using structured, pre-tested and validated questionnaires. The survey also follows similar standard procedure in development of questionnaires, sampling, data collection, and coding which makes multi-country analysis possible. The DHS surveys employs a stratified two-stage cluster sampling technique. In the first stage, clusters/enumeration areas (EAs) that cover the entire country were randomly selected from the sampling frame (i.e. are usually developed from the available latest national census). The second stage, the systematic sampling was employed on households listed in each cluster or EA and interviews were conducted in selected households with target populations (women aged 15–49 and men aged 15–64). In this study, a total weighted sample of 172,774 reproductive aged women who had given birth within the five years preceding the survey of each country were included. For those women who had given more than one birth within five years preceding the survey, the most recent birth was used. In addition, the reproductive aged women with missing value of the outcome variable were excluded from the study (Table 1).

**Table 1:** Countries and survey year of Demographic and Health Surveys included in the analysis for 36 sub-Saharan African countries

Country	Survey year
Angola	2015/16
Burkina Faso	2010
Benin	2017/18
Burundi	2016/17
Central Democratic Congo	2013/14
Congo	2011/12
Cote d’vore	2011/12
Cameroon	2018
Ethiopia	2016
Gabon	2012
Ghana	2014
Gambia	2013
Guinea	2018
Kenya	2014
Comoros	2012
Liberia	2013
Lesotho	2014
Madagascar	2008/09
Mali	2018
Malawi	2015/16
Mozambique	2011
Nigeria	2018
Niger	2012
Namibia	2013
Rwanda	2014/15
Sera lone	2013
Senegal	2010
Sao tome and principe	2008/09
Eswatini	2006/07
Chad	2014/15
Togo	2013
Tanzania	2015/16
Uganda	2016
South Africa	2016
Zambia	2018
Zimbabwe	2015

### Variables of study

#### Dependent variable

The outcome variable for this study was preterm birth among reproductive aged women who had given birth within five years preceding the surveys. This variable was derived from the DHS question, “duration of pregnancy (b20)”. Duration of pregnancy was dichotomized as “Yes” for preterm birth if it ranges or birth was given between 5 to 8 completed months of gestational age, and “No” if it ranges or birth was given after nine or more completed months of gestational age.

#### Independent variables

In this study, individual and community level variables were considered. Women’s age (< 20, 20–34, and > 35), education (no, primary, secondary, and higher), marital status (single, married, divorced and widowed), wealth index (poorest, poorer, middle, richer and richest), currently working status (no and yes), mass media exposure (exposed and not exposed; mothers were considered as exposed to mass media when they watch either television or radio at least once per week and otherwise not exposed (30)), substance use (no, yes), preceding birth interval in months (first birth, < 24, 24–59, and ≥ 60 months), number of ANC visit (< four and ≥ four visits), sex of fetus (male and female), multiple pregnancy (no and yes), contraceptive use (no and yes), history of terminated pregnancy (no and yes), wanted pregnancy (no and yes), pervious delivery by cesarean section (no and yes) were included as the individual level variables of the study. Whereas residence (urban and rural) and regions of Africa (central, east, south and west Africa) were included as the community level variables.

### Data management and statistical analysis

The variables of the study were extracted, cleaned and recoded using STATA version 14. The data were weighted using sampling weight, primary sampling unit, and strata during any statistical analysis to adjust for unequal probability of selection due to the sampling design used in DHS data. Hence, the representativeness of the survey results was ensured.

A two-level multivariable binary logistic regression analysis was used to estimate the effect of explanatory variables on preterm birth among reproductive aged women. The data has two levels with a group of J EAs and within-group j (j = 1, 2…, J), a random sample nj of level-one units (reproductive aged women). The response variable was denoted by;

$${\mathrm{Y}}_{\mathrm{i}\mathrm{j}}=0\ \mathrm{if}\ \mathrm{the}\ {\mathrm{i}}^{\mathrm{th}}\ \mathrm{mother}\ \mathrm{was}\ \mathrm{in}\ \mathrm{the}\ {\mathrm{j}}^{\mathrm{th}}\ \mathrm{EA}'\mathrm{s}\ \mathrm{had}\ \mathrm{delivered}\ \mathrm{term}\ \mathrm{and}\ \mathrm{post}\ \mathrm{term}\ \mathrm{baby}$$

$$1\ \mathrm{if}\ {\mathrm{i}}^{\mathrm{th}\ \mathrm{mother}}\ \mathrm{was}\ \mathrm{in}\ \mathrm{the}\ {\mathrm{j}}^{\mathrm{th}}\ \mathrm{EA}'\mathrm{s}\ \mathrm{had}\ \mathrm{delivered}\ \mathrm{preterm}\ \mathrm{baby}$$

So, appropriate inference and conclusions from this data require advanced modeling techniques like multilevel modeling, which contain variables measured at different levels of the hierarchy, to account the nested effect [33]. Four models were fitted for the data. The first model was an empty model without any explanatory variables, to calculate the extent of cluster variation on preterm birth among reproductive aged women. Variation between clusters (EAs) were assessed by computing Intra-class Correlation Coefficient (ICC), Proportional Change in Variance (PCV) and Median Odds Ratio (MOR). The ICC is the proportion of variance explained by the grouping structure in the population. It was computed as: ICC= $\frac{{\sigma_{\mu}}^2}{{\sigma_{\mu}}^2+\pi^2\left/3\right.}$; Where: the standard logit distribution has variance of $\uppi^2\left/3\right.$, *σ*_*μ*_^2^ indicates the cluster variance. Whereas PCV measures the total variation attributed by individual and community level factors in the multilevel model as compared to the null model. It was computed as: $\frac{\mathrm{variance}\ \mathrm{of}\ \mathrm{null}\ \mathrm{model}-\mathrm{variance}\ \mathrm{of}\ \mathrm{full}\ \mathrm{model}}{\mathrm{variance}\ \mathrm{of}\ \mathrm{null}\ \mathrm{model}}$ [34]. The MOR is defined as the median value of the odds ratio between the cluster at high risk and cluster at lower risk of preterm birth when randomly picking out two clusters (EAs). It was computed as:MOR = exp. ($\sqrt{\ 2\ast {\upsigma_{\upmu}}^2\ast 0.6745\ }$) ~ MOR = exp. (0.95 ∗ σ_μ_) [35]. The second model was adjusted for community level only; the third model was adjusted for individual level variables only while the fourth was fitted with both individual and community level variables. These four models were compared by using deviance (−2LLR) and a model with the lowest deviance was selected as the best-fitted model for the data.

Variables with a *p*-value ≤0.2 in the bi-variable analysis were considered for the multivariable analysis. In the multivariable multilevel binary logistic model, variables with *p* value< 0.05 were declared as statistically significant factors of preterm birth. The Adjusted Odds Ratio (AOR) with 95% Confidence Interval (CI) of the best fitted model were reported to identify the associated factors of preterm birth.

### Ethical consideration

This study is a secondary data analysis from the DHS data, so it does not require ethical approval. For conducting this study, online registration and request for measure DHS were conducted. The dataset was downloaded from DHS on-line archive (http:/www.dhsprogram.com) after getting approval to access the data. All methods were carried out in accordance with the Declaration of Helsinki.

## Results

### Background characteristics

A total of 172,774 reproductive aged women were included in the study, of those 85,064 (49.23%) were in East Africa. Majority of the participants, 121,224 (70.16%) were rural dwellers. Six thousand sixteen (37.63%) participants were not educated. More than two third of the study participants, 122,256 (70.76%) were in the age range of 20–34 years. Majority of the participants, 151,912 (87.92%) were married. Moreover, 96,962 (56.12%) had not exposure to mass media. Regarding to obstetrics history of the participants, 22,274 (12.89%) participants had history of terminated pregnancy, 118,179 (68.40%) were not using any contraceptive method, 28,300 (18.08%) had short birth interval, 9685 (6.52%) pregnancies were not wanted or planned, and 14,159 (13.94%) participants had no ANC visit (Table 2).

**Table 2:** Background characteristics of the reproductive aged women from Sub-Saharan African countries

Variables	Weighted frequency	Percentage (%)
**Region**		
Central Africa	15,773	9.13
East Africa	85,064	49.23
South Africa	3993	2.31
West Africa	67,945	39.33
**Residence**		
Urban	51,551	29.84
Rural	121,224	70.16
**Age (years)**		
< 20	8355	4.84
20–35	122,256	70.76
> 35	42,164	24.40
**Education status**		
No	65,016	37.63
Primary	59,436	34.40
Secondary	41,279	23.89
higher	7045	4.08
**Marital status**		
Single	8734	5.05
Married	151,912	87.92
Divorced	9680	5.60
widowed	2450	1.42
**Wealth index**		
Poorest	39,410	22.81
Poorer	38,144	22.08
Middle	34,889	20.19
Richer	32,274	18.68
Richest	28,059	16.24
**Current working**		
No	58,915	34.10
Yes	113,860	65.90
**Media exposure**		
No	96,962	56.12
Yes	75,813	43.88
**Substance use**		
No	161,851	99.03
Yes	1583	0.97
**Preceding birth interval (months)**		
first birth	21,550	13.77
Short	28,300	20.97
Optimum	89,375.908	57.10
Long	17,299.056	11.05
**Number of ANC visit**		
1–3 visits	117,105.51	67.78
≥ 4 visits	55,668.192	32.22
**Sex of fetus**		
Male	87,479	50.63
Female	85,296	49.37
**Multiple pregnancy**		
No	166,661	96.46
yes	|6113	3.54
**Contraceptive use**		
no	118,179	68.40
Yes	54,596	31.60
**History of terminated pregnancy**		
No	150,500	87.11
Yes	22,274	12.89
**Wanted pregnancy**		
No	9685	6.52
Yes	138,870	93.48
**Pervious delivery by CS**		
No	158,427	95.21
Yes	7978	4.79
**Parity**		
Primi	21,550	12.47
Multi	107,674	62.32
Grand	43,551	25.21

### The pooled prevalence of preterm birth among reproductive aged women in SSA

**Fig. 1:**
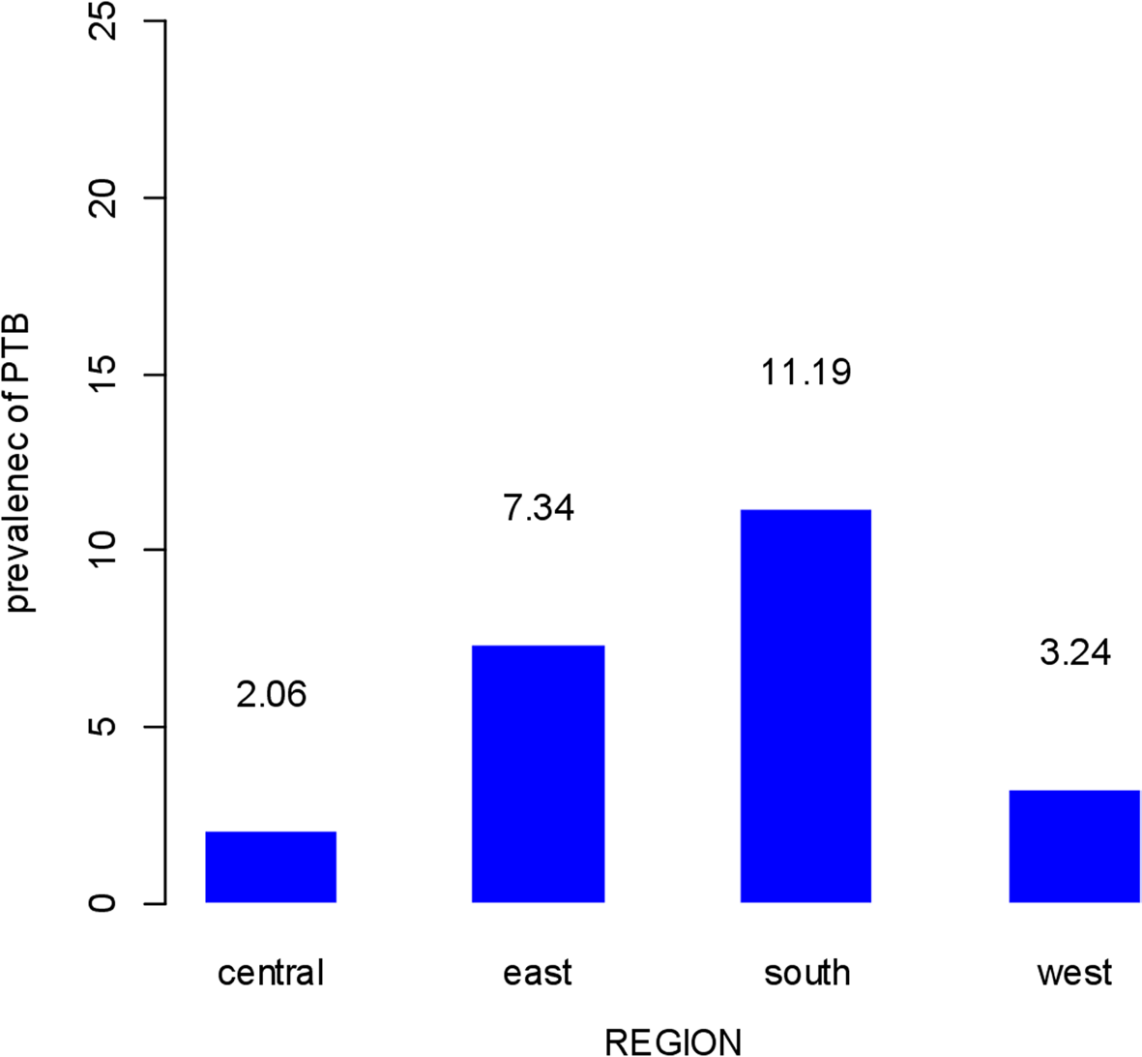
The prevalence of preterm birth in regions of Sub-Sharan African countries

The pooled prevalence of preterm birth among reproductive aged women was 5.33% (95%CI, 5.23, 5.44%) in Sub-Sharan African countries. Central Africa had the lowest prevalence of preterm birth (2.06%) whereas the highest prevalence was founded in South Africa region (11.19%) (Fig. 1) (Supplementary file 1).

### Random effect analysis and model comparison

In first model (empty model), the ICC indicated that about 9.9% of the total variability for preterm birth was due to differences between clusters/EA, with the remaining unexplained 91.1% was attributable to the individual differences. In addition, the median odds ratio also revealed that preterm birth was heterogeneous among clusters. It was 1.41 in the first model which implies reproductive aged women within cluster of having higher risk for preterm birth had 1.41 times higher chance of delivering preterm baby as compared with reproductive aged women within cluster of having lower risk if the women were selected randomly from two different clusters (EAs). Regarding to PCV, about 47.22% of the variability in preterm birth was explained by the full model. Model IV was selected as the best fitted model (had the lowest deviance) (Table 3).

**Table 3:** Multilevel analysis of factors associated with preterm birth among reproductive age women from Sub-Saharan African countries

Variables	Model 1	Model 2	Model 3	Model 4
		AOR with 95% CI	AOR with 95% CI	AOR with 95% CI
**Residence**				
Urban		1		1
Rural		1.21 (1.15 1.27)		1.13 (1.04, 1.22) *
**Region**				
central Africa		1		1
east Africa		4.6 (4.06, 5.10)		4.88 (4.25, 5.61)*
South Africa		7.30 (6.4 8.46)		1.83 (1.58, 2.12)*
West Africa		1.77 (1.57, 1.99)		1.17 (0.89, 1.32)
**Age (years)**				
< 20			1.03 (0.92, 1.15	1.12 (1.01, 1.25)*
20–34			1	1
> 34			0.95 (0.91, 1.06)	0.96 (0.92, 1.07)
**Education**				
No			1	1
Primary			2.40 (2.25, 2.56)	1.53 (1.43, 1.65)*
Secondary			1.73 (1.59, 1.89)	1.36 (1.25, 1.49) *
higher			1.75(1.49, 1.2.05)	1.42 (1.21, 1.67) *
**Marital status**				
Single			1	**1**
Married			0.91 (0.85, 0.97)	0.89 (0.84, 0.93) *
Divorced			1.29 (1.08,1.49)	1.07 (0.93, 1.24)
widowed			1.09 (0.86, 1.39)	0.94 (0.74, 1.28)
**Wealth index**				
Poorest			1	1
poorer			0.70 (0.65, 0.76)	0.75 (0.69, 0.81) *
Middle			0.65 (0.59, 0.70)	0.69 (0.64, 0.76) *
richer			0.74 (0.68, 0.81)	0.75 (0.69, 0.82) *
Richest			0.88 (0.80, 0.97)	0.82 (0.73, 0.91)*
**Current working**				
No			1	1
Yes			1.11 (1.05, 1.18)	1.12 (1.05, 1.18)*
**Media exposure**				
No			1	1
Yes			1.04 (0.99, 1.11)	1.03 (0.96, 1.12)
**Substance use**				
No			1	1
Yes			1.25 (1.18, 1.36)	1.27 (1.09, 1.63) *
**Preceding birth interval (months)**				
First birth			2.04 (1.89, 2.21)	1.88 (1.74, 2.05) *
< 24 months			1.14 (1.06, 1.22)	1.21 (1.13, 1.31) *
24–59 months			1	1
> 60 months			1.07 (0.98, 1.17)	1.01 (0.92, 1.11)
**Number of ANC visit**				
< 4 visits			1	1
≥ 4 visits			0.78 (0.74, 0.82)	0.78 (0.74, 0.83) *
**Sex of fetus**				
Male			1	1
Female			1.08 (1.03, 1.15)	1.08 (1.03, 1.15) *
**Multiple pregnancy**				
No			1	
Yes			3.18 (2.87, 3.52)	3.37 (3.04, 3.74)*
**Terminated pregnancy History**				
No			1	1
Yes			1.24 (1.16, 1.34)	1.21 (1.13, 1.31) *
**Wanted pregnancy**				
No			1	1
Yes			0.65 (0.59, 0.71)	0.75 (0.68, 0.83) *
**Pervious delivery by CS**				
No			1	1
Yes			1.62 (1.44, 1.84)	1.46 (1.29, 1.65) *
Community level variance	0.36	0.32	0.23	0.19
ICC (%)	9.9%	8.8%	6.5%	5.5%
MOR	1.41	1.34	1.24	1.19
PCV (%)	reference	11.11%	36.11%	47.22%
Deviance	72,624	70,337	47,244	45, 945

*indicates *p*-value <=0.05

#### Factors associated with preterm birth

In the bi-variable multilevel analysis, all of the explanatory variables (both individual level and community level variables) except contraceptive use had shown statistically significant association with preterm birth at a *p*-value of < 0.20.

In the final model, community level variables such as region and residence, and individual level variables like women’s age, education, marital status, wealth index, current working status, substance use, number of ANC visit, multiple birth, history of terminated pregnancy, wanted pregnancy, preceding birth interval, and previous delivery by CS were significantly associated with preterm birth (*p* ≤ 0.05).

The study revealed that the odds of having preterm birth was 4.88 (AOR = 4.88, 95%CI:4.25, 5.61) and 1.83 (AOR = 1.83, 95%CI:1.58, 2.12) times for East and South Africa regions as compared to central region, respectively. Besides, the likelihood of having preterm birth was 12% (AOR = 1.12, 95%CI:1.01, 1.25) higher for teenagers as compared to women aged from 20 to 34 years. The odds of delivering preterm baby among reproductive aged women was 13% (AOR = 1.13; 95%CI: 1.04, 1.22) higher for women living in rural areas compared to their counterparts. As compared with women in the poorest wealth index, the odds of having preterm birth was 25% (AOR = 0.75, 95%CI: 0.695, 0.81), and 31% (AOR = 0.69, 95%CI: 0.64, 0.76), 25% (AOR = 0.75, 95%CI: 0.69, 0.82), 18% (AOR = 0.82, 95%CI: 0.73, 0 .91) lower for mothers having poorer, middle, richer and richest, respectively. Regarding maternal education, the odds of having delivering preterm baby was 1.53 (AOR = 1.53, 95%CI: 1.43, 1.65), 1.36 (AOR = 1.36, 95%CI: 1.25, 1.49), 1.42 (AOR = 1.42, 95%CI: (1.21, 1.67) times higher for women’s having primary, secondary and higher education as compared to those with no education, respectively. Married women had 11% (AOR = 0.89, 95%CI: 0.84, 0.93) lower chance of having preterm birth as compared to single women. This study also revealed that the odds of having preterm birth among women that were currently working was 1.12 (AOR = 1.12, 95%CI: 1.05, 1.18) times to their counterparts. Female fetus had 8% (AOR = 1.08, 95%CI: 1.03, 1.15) higher chance to be preterm birth as compared to male. Besides, babies born from mothers who were using substance had 27% (AOR = 1.27, 95%CI: 1.094, 1.63) higher chance of having preterm birth as compared to their counterparts.

The odds of preterm birth were increased by obstetrical factors such as multiple pregnancy (AOR = 3.37, 95%CI: 3.04, 3.74), history of terminated pregnancy (AOR = 1.21, 95%CI:1.13, 1.31), and previous delivery by CS (AOR = 1.80, 95%CI:1.29, 1.65). As compared to women’s having optimum preceding birth interval, the odds of having preterm birth was 1.88 (AOR = 1.88, 95%CI:1.74, 2.05) and 1.21 (AOR = 1.21, 95%CI: 1.13, 1.31) times higher for women giving their first birth and who had short preceding birth interval, respectively. Moreover, women’s having four and above ANC visits (AOR = 0.0.78, 95%CI: 0.74, 0.83), and the pregnancy was wanted (AOR = 0.75, 95%CI: 0.68, 0.83) were the important obstetrical factors that reduce the chance of preterm birth (Table 3).

## Discussion

This study aimed to assess the pooled prevalence and associated factors of preterm birth in Sub-Saharan Africa countries using their recent DHS data. According to the findings of this study, preterm birth was a significant public health problem (5.33%) in SSA. The higher burden of preterm birth in this study was in line with studies conducted in different parts of the world [36–40]. This could be explained by low maternal health services utilization such as ANC, short birth intervals, and engaging in heavy activities like agriculture [41, 42].

In this study, we found that the risk of preterm birth was higher for teenager as compared with women aged from 20 to 34 years. This finding was supported by previous study [43]. The highest risk for preterm birth for teenager’s might be related with that most teenage girls may not be matured enough for pregnancy and childbearing. On the other hand, the pregnancy in this age group are usually unplanned or unintended which could result in less interest and chances of mother to seek prenatal and antenatal cares. Besides, young women are more exposed to many risky behaviors like substance use and less adherence to counseling and education given by their healthcare providers compared to older women. This conditions could have led to adverse outcomes of pregnancy.

In consistent with previous studies [44, 45], women from rural areas had higher risk of preterm birth as compared with their counterparts. The possible explanation for this finding could be women in rural areas had poor ANC attendance and they usually experience unwanted pregnancy [46, 47]. In addition, this woman had poor access, availability, and utilization of health care services compared to women living in urban areas [48]. Education was also the significant and important factor of preterm birth among reproductive aged women. The odds of delivering preterm babies were higher for educated mothers as compared with not educated. This finding is in contradict with previous studies conducted in different parts of the world [19, 49, 50]. This could be linked with the educated mothers had higher chance of having previous CS delivery and usually work actively during their pregnancy which are the major risk factors for premature delivery [23, 51].

This study also revealed that the babies from married women had lower risk to be delivered prematurely than from single women. This finding was supported by previous study done on different areas of the world [21, 37]. This might be related to good financial security, low psychosocial stress and better health care utilization among married women that directly or indirectly affects the occurrence of preterm birth [52].

This study identified that short preceding birth interval (< 24 months) increases the risk of delivering preterm babies. This finding was in agreement with previous studies [12, 53, 54]. The possible reason for this finding could be mothers with short preceding birth interval do not have time to recover from the physical stress and nutritional burden of the pregnancy and child birth [55]. In agreement with previous studies, the risk of delivering preterm birth were higher for women giving their first birth [56]. The women who are going to deliver their first baby are at increased risk for several medical and obstetrical complications such as hypertensive disorders of pregnancy [57]. Thus, Women with such complications are often delivered by a pre-labor induction or cesarean section at preterm gestational age in order to minimize adverse pregnancy outcomes [58].

The chance of giving preterm birth was also higher among women delivering multiple pregnancy and women having previous history of CS delivery. This finding was supported by a previous study conducted in Saudi [59]. This could be due to the greater fetal size, larger placental and amniotic fluid volumes in multiple pregnancies that stimulates the physiology of labor earlier than singleton pregnancies [31].

The chance of delivering preterm birth was higher among women that have history of terminated pregnancy (abortion and stillbirth) compared to their counterparts. The finding of this study was also supported by previous studies done in different parts of the world [60, 61]. The reason for this association could be explained by history of terminated pregnancy and preterm birth may share common biological, genetic and environmental factors [62].

According to this study, the chance of delivering preterm birth was lower for mothers that had wanted pregnancy. This finding was in agreement with previous studies conducted in elsewhere [63, 64]. This might be due to women with unwanted pregnancies were more likely to smoke cigarettes, use illicit drugs, consume alcohol during pregnancy, and less likely to get ANC services during their pregnancy [65, 66]. This could directly or indirectly affect the occurrence of preterm birth among mothers giving birth.

This study revealed that the risk of preterm birth was higher for women’s who uses substance as compared to their counterparts. The finding of this study was supported by studies done in different parts of the world [67–71]. This finding could be related with alcohol consumption and substance use are a potential risk for congenital anomalies [72, 73]. A pregnancy with a fetus having congenital anomalies had high risk of adverse pregnancy outcomes including preterm birth [74]. Moreover, substance users such as tobacco or cigarette, and those who drink alcohol had experienced unwanted/unplanned pregnancy that usually end up with abortion [75, 76].

Currently working status was also significantly and independently associated with preterm birth. The odds of delivering preterm baby for currently working mother was higher as compared to their counterparts. The finding of this study was in consistent with previous findings [77, 78]. This could be better explained by currently working women might have hazardous occupational exposure during their prenatal and pregnancy period [79]. This conditions affect their pregnancy and might end up with adverse outcomes.

### Strength and limitation of the study

The main strength of this study was the use of weighted, pooled national representative large datasets of SSA countries with the use of advanced statistical analysis technique that accounts the correlated nature of DHS data, which enables us more precise estimates and standard errors. However, this study is not free from limitations. Since, the DHSs are cross-sectional surveys, we cannot establish a cause-and-effect relationship between the different independent variables and Preterm birth. Moreover, since the data were collected through interview, there might be a possibility of recall bias because women may forget the exact duration pregnancy. On the other hand, this study had faced misclassification bias because the cut points for preterm births in weeks that ranges from 28 to 37 but the data was collected in moths. This situation leds to categorization of preterm babies as term baby.

## Conclusion

Delivering preterm baby among reproductive aged women in SSA remains a major public health problem. Residence, education, marital status, wealth index, currently working status, and substance use and obstetrical factors like number of ANC visit, multiple pregnancy, history of terminated pregnancy, wanted pregnancy, preceding birth interval, and previous delivery by CS were the associated factors of preterm birth. Therefore, it is better to consider women with terminated pregnancy, previous caesarian delivery, multiple pregnancy, short birth interval, and giving their first birth during intervention to prevent the short-term and long-term consequences of preterm birth.

## Supplementary Information

**Additional file 1: Supplementary file 1**: Cross-tabulation of preterm birth in different socio-economic and obstetrical factors from 36 Sub-Saharan African countries.

## Data Availability

The datasets used and/or analyzed for this study are available from the Demographic and Health Surveys (DHS) Program (https://dhsprogram.com/Data/).

## Abbreviations

AOR: Adjusted Odds Ratio
ANC: Antenatal care
AOR: Adjusted Odds Ratio
CDI: Community Development Index
ICC: Intra-class Correlation Coefficient
CI: Confidence Interval
DHS: Demographic and Health Survey
MOR: Median Odds Ratio
MM: Mass Media exposure
WHO: World Health Organization
