# Supplementary material for: Preterm birth and its associated factors among reproductive aged women in sub-Saharan Africa: evidence from the recent demographic and health surveys of sub-Sharan African countries
Source: BMC Pregnancy Childbirth. 2021 Nov 15;21:770. doi: 10.1186/s12884-021-04233-2 (PMC8591945; doi:10.1186/s12884-021-04233-2)
Supplement: Supplementary file 1 — Additional file 1: Supplementary file 1: Cross-tabulation of preterm birth in different socio-economic and obstetrical factors from 36 Sub-Saharan African countries. [file 12884_2021_4233_MOESM1_ESM.docx]

Supplementary file 1: cross-tabulation of preterm birth in different socio-economic and obstetrical factors from 36 Sub-Saharan African countries

| **Variables** | **Category** | **Preterm** | |
| --- | --- | --- | --- |
|  |  | **no** | **yes** |
| **Residence** | Urban | 48,797 | 2,754 |
|  | Rural | 114,766 | 6,458 |
| **Region** | Central Africa | 15,449 | 325 |
|  | east Africa | 78,824 | 6,241 |
|  | South Africa | 3,547 | 447 |
|  | West Africa | 65,74 | 2,201 |
| **Age (years)** | <20 | 7,686 | 670 |
|  | 20-35 | 115,441 | 6,815 |
|  | >35 | 40,436 | 1,728 |
| **Education** | No | 62,973 | 2,043 |
|  | Primary | 5,159 | 4,278 |
|  | Secondary | 38,834 | 2,445 |
|  | higher | 6,598 | 448 |
| **Marital status** | Single | 7,953 | 781 |
|  | Married | 144,393 | 7,519 |
|  | Divorced | 8,891 | 789 |
|  | widowed | 2,327 | 124 |
| **Wealth index** | Poorest | **37,124** | **2,287** |
|  | poorer | 36,349 | 1,796 |
|  | Middle | 33,266 | 1,623 |
|  | richer | 30,579 | 1,695 |
|  | Richest | 26,248 | 1,812 |
| **Current working** | No | 55,999 | 2,917 |
|  | Yes | 107,565 | 6,295 |
| **Media exposure** | No | 92,313 | 4,650 |
|  | Yes | 71,251 | 4,563 |
| **Substance use** | No | 153,141 | 92 |
|  | Yes | 1,492 | 8,710 |
| **Preceding birth interval (months)** | First birth | 19,794 | 1,756 |
|  | < 24 months | 26,932 | 1,368 |
|  | 24-59 months | 85,524 | 3,853 |
|  | > 60 months | 16,388 | 912 |
| **Number of ANC visit** | <4 visits | 110,648 | 6,458 |
|  | ≥ 4 visits | 52,915 | 2,754 |
| **Sex of fetus** | Male | 82,970 | 4,509 |
|  | Female | 80,594 | 4,703 |
| **Multiple pregnancy** | No | 158,369 | 8,292 |
|  | Yes | 5,194 | 920 |
| **terminated pregnancy History** | No | 142,661 | 7,840 |
|  | Yes | 20,902 | 1,372 |
| **Wanted pregnancy** | No | 9,004 | 680 |
|  | Yes | 131,575 | 7,295 |
| **Pervious delivery by CS** | No | 152,712 | 8,314 |
|  | Yes | 5,668 | 612 |
